# Pesticide exposure and risk of Alzheimer’s disease: a systematic review and meta-analysis

**DOI:** 10.1038/srep32222

**Published:** 2016-09-01

**Authors:** Dandan Yan, Yunjian Zhang, Liegang Liu, Hong Yan

**Affiliations:** 1 Department of Health Toxicology, MOE Key Lab of Environment and Health, School of Public Health, Tongji Medical College, Huazhong University of Science and Technology, 13 Hangkong-Road, Wuhan, 430030, PR China; 2 Department of Neurology, Union Hospital, Tongji Medical College, Huazhong University of Science and Technology, 1277 Jiefang Avenue, Wuhan, 430022, PR China; 3 Department of Nutrition and Food Hygiene, Hubei Key Laboratory of Food Nutrition and Safety, Tongji Medical College, Huazhong University of Science and Technology, 13 Hangkong-Road, Wuhan, 430030, PR China

## Abstract

Evidence suggests that lifelong cumulative exposure to pesticides may generate lasting toxic effects on the central nervous system and contribute to the development of Alzheimer’s disease (AD). A number of reports indicate a potential association between long-term/low-dose pesticide exposure and AD, but the results are inconsistent. Therefore, we conducted a meta-analysis to clarify this association. Relevant studies were identified according to inclusion criteria. Summary odds ratios (ORs) were calculated using fixed-effects models. A total of seven studies were included in our meta-analysis. A positive association was observed between pesticide exposure and AD (OR = 1.34; 95% confidence interval [CI] = 1.08, 1.67; n = 7). The summary ORs with 95% CIs from the crude and adjusted effect size studies were 1.14 (95% CI = 0.94, 1.38; n = 7) and 1.37 (95% CI = 1.09, 1.71; n = 5), respectively. The sensitivity analyses of the present meta-analysis did not substantially modify the association between pesticide exposure and AD. Subgroup analyses revealed that high-quality studies tended to show significant relationships. The present meta-analysis suggested a positive association between pesticide exposure and AD, confirming the hypothesis that pesticide exposure is a risk factor for AD. Further high-quality cohort and case-control studies are required to validate a causal relationship.

Alzheimer’s disease (AD) is the most common progressive neurological disease and results in an irreversible loss of neurons^1^. Late-onset AD, which typically develops after age 60, is the most common form and is characterized by the insidious onset of dementia, progressive memory loss, and cognitive decline ultimately leading to dysfunction in daily life and work abilities^2^. Pathologically, amyloid plaques and neurofibrillary tangles are the main forms of aggregated proteins involved in AD. Amyloid plaques are visible protein aggregates derived from the dimers and oligomers of brain amyloid-β protein, while neurofibrillary tangles are composed of a compact filamentous network of helical filaments of hyperphosphorylated tau protein. Together these neuropathological changes are thought to result in the loss of synapses and neuronal cell death, leading in cognitive dysfunction^3^,^4^.

The aetiology of late-onset AD remains largely unknown but is believed to be multifaceted, resulting from both genetic and environmental factors^5^. The main risk factor is clearly age, though research has identified some genetic risk factors that may account for a small percentage of AD cases^6^. The most well established genetic component of AD risk is the APOE-ε4 allele^7^,^8^. Epidemiological studies have reported a higher prevalence of AD in rural areas than in urban settings. Over the past decades, pesticides have been used to increase the productivity and the specialization of cultures in rural areas^9^. Evidence from *in-vitro* models and animal studies suggests that long-term/low-dose pesticide exposure may lead to the neuronal loss in specific brain regions, resulting in subsequent cognitive impairment, decreased memory and attention, and loss of motor function^10^,^11^. These neurobehavioral dysfunctions may ultimately lead to AD and other forms of dementia later in life^12^,^13^.

Pesticides are known neurotoxins. Although the mechanisms underlying the influence of pesticides on neurodegenerative disease remain to be elucidated, the role of long-term/low-dose exposure to pesticides such as paraquat, dieldrin, organochlorine and organophosphates has long been suspected. Most pesticides share a number of features, such as the ability to induce oxidative stress, mitochondrial dysfunction, α-synuclein fibrillization and neuronal loss^14^. Furthermore, the use of pesticides has increased dramatically in the last 50 years, which may present a potentially important public health issue^15^,^16^.

In order to investigate the epidemiological relationship between AD and pesticide exposure, we performed a comprehensive systematic review and meta-analysis of published cohort and case-control studies on the association between pesticide exposure and development of AD.

## Results

### Search results

**Figure 1:**
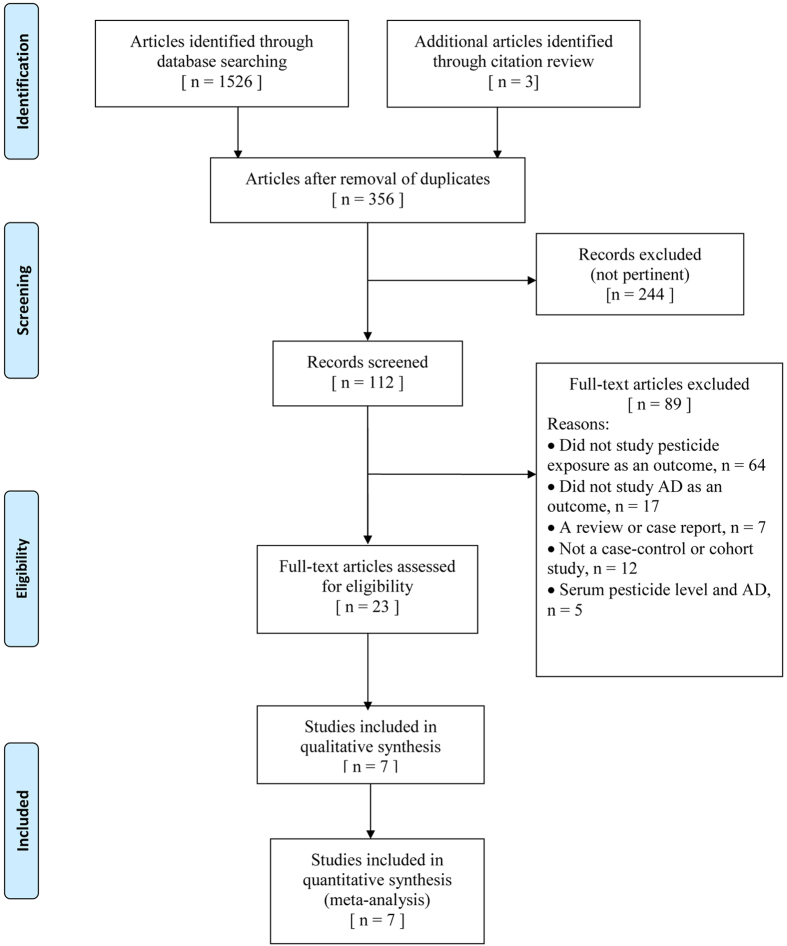
Flow diagram of systematic review of literature concerning pesticide exposure and Alzheimer’s disease (AD) risk.

Our initial search of all databases retrieved 1,526 studies; however many of these were duplicates. After review of titles and abstracts, we identified 104 pertinent articles. We excluded reviews, editorials, nonhuman studies, and case reports; we also excluded publications that explored the association between serum pesticide levels and risk of AD, as these data could not be meta-analysed because of their different formats in the reporting of results. After excluding those studies that failed to meet the inclusion criteria, a total of seven publications were selected, providing data on three cohort^12^,^17^,^18^ and four case-control studies^19^,^20^,^21^,^22^. The flowchart of literature selection is shown in Fig. 1.

### Study characteristics

The seven eligible studies included 6,835 participants, of which 1,050 were AD patients. The main characteristics of the selected studies are presented in Table 1. The results of quality assessment conducted according to the Newcastle-Ottawa Scale are shown in Supplementary Table S1 (available online). The estimated scores of all included studies were in the range of 5–8 points.

### Meta-analysis

**Figure 2:**
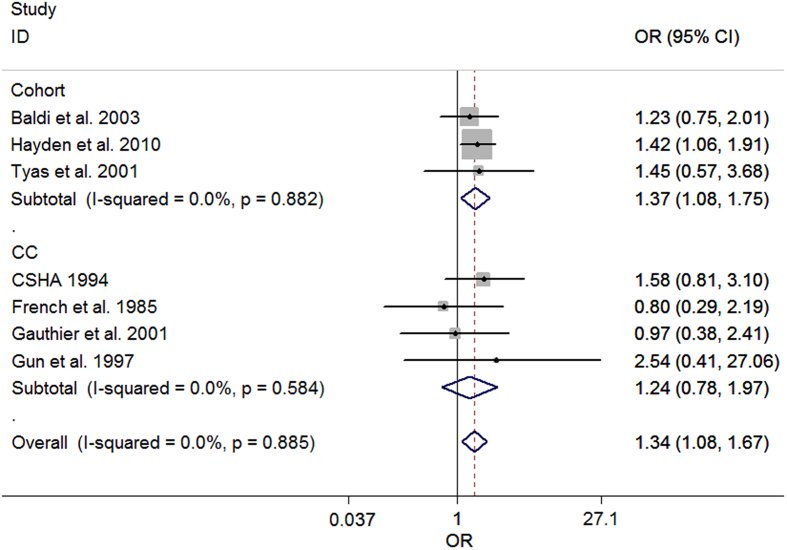
Forest plot of pesticide exposure and risk of AD. The points and horizontal lines correspond to the study-specific odds ratios (ORs) and 95% confidence intervals (CIs), respectively. The grey areas reflect the study-specific weight. The diamonds represent the pooled ORs and 95% CIs. The vertical dashed line indicates an OR of 1.34.

Heterogeneity between the included studies did not exceed that expected by chance (*P* = 0.88 and *I^2^* = 0.0%), indicating that the results across the selected articles were statistically homogeneous. We used a fixed-effects model to calculate summary odds ratios (ORs) and 95% confidence intervals (CIs). The summary OR of pesticide exposure and AD was 1.34 (95% CI = 1.08, 1.67), suggesting a significantly positive association, the forest plot was shown in Fig. 2. The summary ORs from the crude effect size and adjusted effect size studies were 1.14 (95% CI = 0.94, 1.38) and 1.37 (95% CI = 1.09, 1.71), respectively. Table 2 summarizes the results of the different format meta-analyses.

### Subgroup analyses

Pesticide exposure was associated with an increased risk of AD in all subgroup analyses, but only half reached statistical significance. In the subgroup of study design, a significantly increased risk was observed in cohort studies (OR = 1.37; 95% CI = 1.08, 1.75), but not in case-control studies (OR = 1.24; 95% CI = 0.78, 1.97). When stratified by method used to assess exposure, the OR of the self-reported group was 1.37 (95% CI = 1.08, 1.75), and the OR of the proxy-reported group was 1.24 (95% CI = 0.78, 1.97).

**Figure 3:**
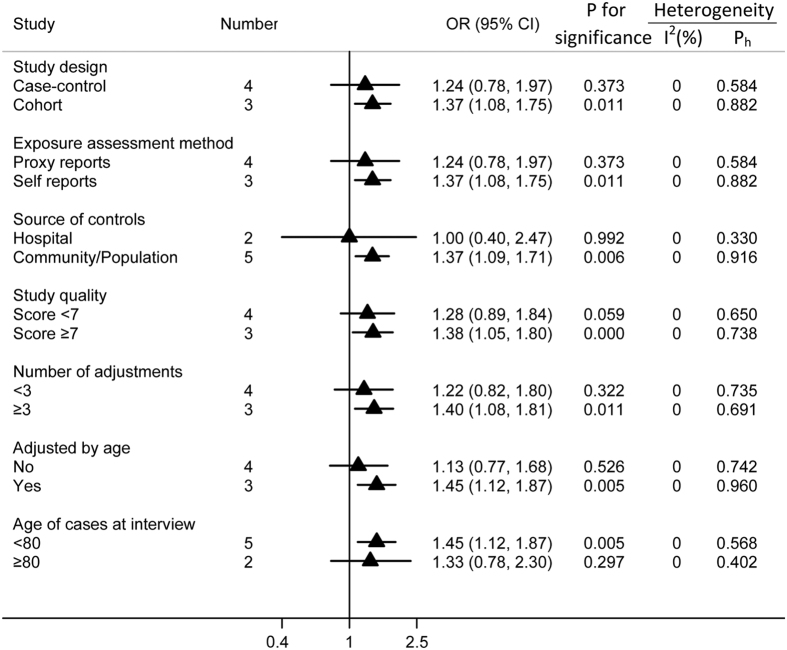
Subgroup analyses of pesticide exposure and risk of AD. The triangles and horizontal lines correspond to the subgroup specific ORs and 95% CIs, respectivly. The vertical solid line indicates an OR of 1. “Ph” represents the *P* value for heterogeneity from the Q-test.

When stratified by control source, no significant association was observed for studies utilizing hospital controls (OR = 1.0; 95% CI = 0.40, 2.47). In contrast, we observed a significant association between pesticide exposure and AD in studies utilizing community and population based controls (OR = 1.37; 95% CI = 1.09, 1.71). When stratified by study quality (score < 7 or ≥7), significant association was observed for study scores ≥7 (OR = 1.42; 95% CI = 1.10, 1.81), though the association observed for study scores < 7 was not significant (OR = 1.13; 95% CI = 0.73, 1.76). Moreover, in order to avoid the residual confounding variable of age, we also conducted subgroup analyses based on whether or not the pooled ORs had been adjusted for age. The results revealed an increased risk in the age-adjusted group (OR = 1.45; 95% CI = 1.12, 1.87), but not in the non-age adjusted group (OR = 1.13; 95% CI = 0.77, 1.68). In addition, pesticide exposure was associated with AD for studies in which the mean age of patients with AD was less than 80 years old (OR = 1.45; 95% CI = 1.12, 1.87), while non-association was noted among studies in which the mean age of patients with AD was greater than 80 years old (OR = 1.33; 95% CI = 0.78, 2.30). Furthermore, pesticide exposure was found to be associated with AD for studies whose results had been adjusted for more than three variables. The results of subgroup analyses are shown in Fig. 3.

### Sensitivity analyses

**Figure 4:**
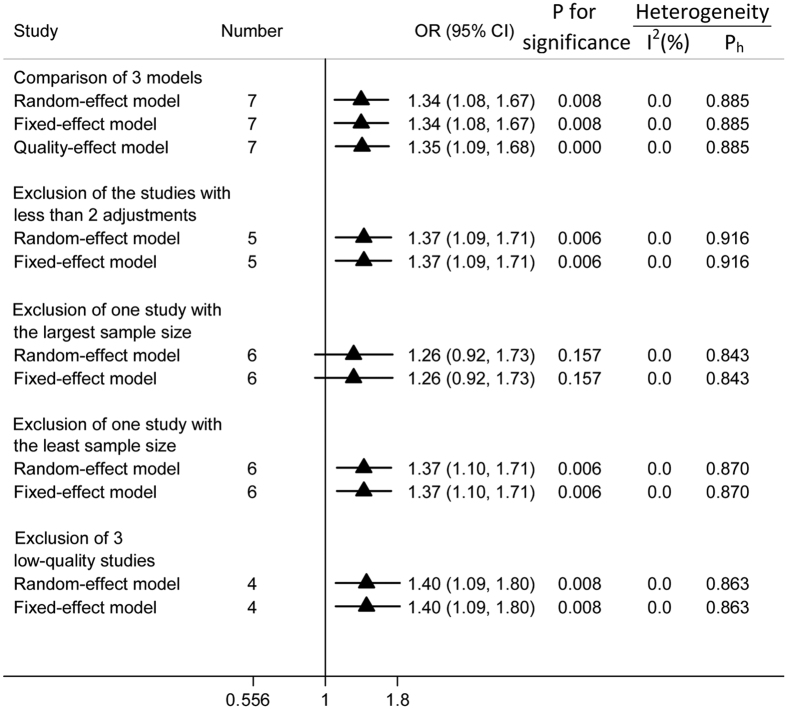
Sensitivity analyses of pesticide exposure and the risk of AD. The triangles and horizontal lines represent the corresponding ORs and 95% CIs. The vertical solid line indicates an OR of 1. “Ph” represents the *P* value for heterogeneity from Q-test.

Sensitivity analyses were conducted to evaluate the sensitivity of our conclusions. First, the random-effect model and fixed-effect model were compared with the quality-effect model, and the conclusions remained unchanged. Second, we omitted each study from the analysis one-by-one (i.e., leave-one-out-method). The conclusion was not drastically changed upon this analysis, and ORs ranged from 1.26 (95% CI = 0.92, 1.73) to1.38 (95% CI = 1.10, 1.71). All results were either significant or of marginal significance. The results of the leave-one-out analysis are shown in Supplementary Figure S1 (available online). Third, specific publications such as studies with fewer than two adjustments, the study with the largest sample size, the study with the smallest sample size, and studies with quality scores less than 7 were excluded successively, and the conclusions remained stable. All analyses were conducted by applying both random-effect and fixed-effect models, and the results of these analyses are displayed in Fig. 4.

### Publication bias

Neither Egger’s test nor Begg’s test suggested any evidence of publication bias for pesticide exposure (Egger, *P* = 0.66; Begg, *P* = 0.76), for studies reporting crude effect size (Egger, *P* = 0.95; Begg, *P* = 1.00), or for studies reporting adjusted effect size (Egger, *P* = 0.55; Begg, *P* = 0.81). Thus, no significant evidence of substantial publication bias was observed in this study.

## Discussion

The relationship between pesticide exposure and risk of AD has attracted an increasing amount of attention in recent years. Pesticides are well-known neurotoxins and are associated with many neurodegenerative disorders, including mild cognitive impairment and dementia, which are strongly linked to AD. Mild cognitive impairment is a prodromal phase of cognitive decline that may precede the emergence of AD. Some research has suggested that mild cognitive impairment and late-onset AD are essentially part of the same pathophysiological process, sharing a number of etiological factors^23^. A prospective cohort study revealed a positive association between pesticide exposure and mild cognitive impairment, suggesting that people with frequent pesticide exposure, such as gardeners and farmers, may have a higher risk of developing AD^24^. In another cohort study^25^, the authors found that participant who had been exposed to pesticides exhibited decreased cognitive performance when compared to a non-exposure group. Moreover, several studies have recognized that chronic or occupational pesticide exposure is a possible risk factor for dementia. For example, one of our selected cohort studies^12^ reported an increased risk for all-cause dementia among individuals who had been exposed to pesticides (HR = 1.38; 95% CI = 1.09, 1.76). In addition, the Canadian Study of Health and Aging found that exposure to occupational pesticides doubles the risk of vascular dementia^26^,^27^. Further evidence regarding the potential role of pesticides in the development of AD has also been reported in both *in vitro* and *in vivo* studies. Several *in vitro* studies have documented that dichlorodiphenyltrichloroethane (DDT) significantly increases levels of amyloid- β precursor protein (AβPP) and β-site AβPP-cleaving enzyme 1(BACE1), impairing the clearance and extracellular degradation of amyloid-β peptides^28^,^29^. An *in vivo* study revealed that some pesticides may disrupt metabolic pathways involved in the homeostasis of amyloid-β, causing a significant increase in amyloid-β levels in the cortex and hippocampus, as well as increased memory loss and reduced motor activity in experimental animals^30^. Thus, some researchers have hypothesized that pesticide exposure is a potential risk factor for AD, and this hypothesis has been further validated by the results of several epidemiological studies^12^,^18^. Because the results of these studies are controversial, however, meta-analysis is an important method that can be used to reveal trends that may not be evident in a single epidemiological study. Thus, it is necessary to conduct a comprehensive systematic review to evaluate the relationship between long-term/low-dose level pesticide exposure and AD risk. However, due to the limited number of original studies and minimal knowledge of the mechanisms underlying the association between pesticide exposure and AD, the relevant meta-analysis has not been performed until now.

To our knowledge, the present study is the first comprehensive meta-analysis combining data from cohort and case-control studies to investigate the possible relationship between pesticides and AD. The results of our analysis suggest a positive association between overall pesticide exposure and AD (OR = 1.34; 95% CI = 1.08, 1.67), without heterogeneity (*P* = 0.88 and *I^2^* = 0.0%), indicating that the selected articles were statistically homogeneous, and that the results exhibited relative reliability. Sensitivity analyses yielded similar results, indicating that the relationships were relatively stable. Moreover, our results were consistent with an ecological study^13^ and several internal pesticide exposure reports^25^,^29^.

The ecological study^13^, involved the selection 17,942 subjects, and used the extent of intensive agriculture and pesticide sales to categorize patients according to their living status in areas of high and low pesticide exposure. The results of this study revealed that the population living in areas with high pesticide exposure had an increased prevalence of AD (OR = 2.10; 95% CI = 1.96, 2.25). Unfortunately, this ecological study could not be included in the present meta-analysis due to the design of study, which explored the prevalence rather than the incidence of AD.

One internal exposure investigation^29^ evaluated the relationship between serum dichlorodiphenyldichloroethylene (DDE) levels and AD, observing a 3.8-fold increase in serum levels of organochlorine metabolites of DDE in patients with AD when compared with control participants. Moreover, in another internal exposure study^31^, the authors found a significant association between AD and high-level exposure to β-hexachlorocyclohexane (OR = 2.06; 95% CI = 1.04, 3.10), and dieldrin (OR = 2.09; 95% CI = 1.22, 3.56). When taken with the results of the aforementioned studies, our findings indicate a positive association between pesticide exposure and AD risk.

Our results revealed a reliable and stable positive association between overall pesticide exposure and AD; however, the evidence is still inconclusive to some extent, as only selected cohort studies exhibited such a positive association, while case-control studies failed to show an association. Generally speaking, cohort studies are preferable to case-control studies for investigating etiological relationships because exposure information from case-control studies is retrospective, which can lead to recall and exposure biases. Thus, further prospective cohort studies are needed to verify this relationship.

The subgroup analyses of the present study indicate that the summary effect sizes from cohort studies revealed a significantly increased risk of AD, while the effect sizes from the case-control studies showed the relationship was null. The reasons are as follows. First, the exposure information in case-control studies was collected from proxy reports (usually from close relatives) due to the mental decline of patients with AD, and this type of information may increase the degree of misclassification bias. Such retrospective data are less reliable, and recall bias becomes unavoidable. Furthermore, the exposure information of both patients control and participants was provided by surrogate respondents in case-control studies; thus, the presence of non-differential bias may result in an underestimation of the effect size^21^. Second, in our meta-analysis, the sample sizes of the case-control studies were much smaller than those of the cohort studies, which would reduce power for statistical significance to some extent. Third, the patients with AD were selected from hospitals in case-control studies; thus, admission rate bias may exist.

In epidemiological studies, adjustments should be conducted to reduce potential confounding variables and achieve more reliable conclusions. Our present study showed that the summary OR of studies reporting adjusted effect sizes was 1.37 (95% CI = 1.09, 1.71), exhibiting a higher risk than those reporting crude effect sizes (OR = 1.14; 95% CI = 0.94, 1.38). In our meta-analysis, age was an important factor associated with the risk of AD and pesticide exposure duration^32^,^33^. The difference between adjusted effect size studies and crude effect size studies may therefore be due to obscure of potential confounds. Moreover, this result also further confirmed the reliability of our findings because the association remained statistically significant even when adjusting for major confounding factors. Furthermore, in our selected studies, most participants were agricultural workers and had been working for most of their lives in agricultural fields where pesticides were widely used. Thus the workers’ long-term/low-dose level exposure to pesticides might generate cumulative neurotoxicity, which could ultimately lead to AD or other neurodegenerative diseases.

Potential bias is the major challenge for meta-analyses of observational studies^34^. In the subgroup analyses of the different methods used to assess levels of exposure, proxy-reported studies (OR = 1.24; 95% CI = 0.78, 1.97) indicated a lower risk than self-reported studies (OR = 1.37; 95% CI = 1.08, 1.75). This difference is likely due to non-differential bias in the proxy-reported group as previously mentioned. When subgroup analyses were conducted according to the source of control participants, study quality, number of adjustments, and age status, the results revealed that studies utilizing community and population-based controls, high-quality studies (scores ≥7), studies with more than three adjustments, and studies that adjusted for age all indicated higher estimated risk than the other subgroups. This difference may result from the more appropriate design of these studies. Specifically, in the group of studies in which the mean age of patients with AD was less than 80 years old, the risk of AD was significant (OR = 1.45; 95% CI = 1.12, 1.87), whereas in the group of studies in which the mean age of patients with AD was greater than 80 years old, the association was not significant (OR = 1.33; 95% CI = 0.78, 2.30). This difference is likely due to the confounding effect of age because AD is a disease of age as mentioned above^33^. Generally, older people have a higher risk of developing AD; thus, the effect of pesticide exposure in older people would be obscured by the effect of age.

The method of AD diagnosis is less likely to be a source of bias in the present study because our included studies used only one criterion^35^ (the National Institute of Neurological and Communicative Disorders and Stroke–Alzheimer’s Disease and Related Disorders Association [NINCDS-ADRDA]). Risk appears to increase as the duration of cumulative exposure increases, as different routes of exposure may produce synergistic effects in increasing risk. However, it was not possible to investigate a dose-response relationship between pesticide exposure and AD or provide a cutoff for exposure in the present meta-analysis.

The highlight of our study is that it is the first comprehensive meta-analysis to investigate the possible relationship between pesticide exposure and the risk of AD. A second advantage is that no heterogeneity was observed among the original studies, suggesting that the result of the present study reveals a reliable relationship. A third advantage is that the sensitivity analysis of our present study did not substantially modify the association between pesticide exposure and AD, indicating stable results. Moreover, our conclusions are consistent with most previous studies, including internal pesticide exposure studies and those regarding the influence of pesticides on dementia and mild cognitive impairment.

However, several limitations exist in the present meta-analysis. First, as we did not search for unpublished studies or original data, publication bias may be inevitable, even though no significant evidence of publication bias was observed. Second, exposure bias was unavoidable because the methods of exposure determination in the original studies ranged from self-administered questionnaires to proxy reports. Third, due to lack of relevant studies, the relationship among AD and duration of pesticide exposure, and patient occupation was not thoroughly investigated. Lastly, we did not thoroughly investigate potential differences with regard to exposure to specific compounds or functional group of pesticide exposure in our subgroup analyses.

The results of our meta-analysis suggest a significant positive association between pesticide exposure and incidence of AD. These findings provide powerful evidence supporting the hypothesis that pesticide exposure is related to an increased risk of AD. Further prospective cohort studies and high-quality case-control studies with improved methods for estimating cumulative pesticide exposure and documenting cases of AD are required to validate the existence of a causal relationship.

## Methods

### Search strategy

We searched PubMed, EMBASE, and Web of Science databases for all English-language cohort and case-control studies published in peer-reviewed journals through April, 2016. PubMed search terms were (“Alzheimer Disease” OR “Dementia” OR “Alzheimer Disease”[Mesh]) AND (“exposure” OR “Occupational Exposure”[Mesh] OR “Environmental Exposure”[Mesh] OR “Pesticides”[Mesh] OR “Herbicides”[Mesh] OR “Fungicides”[Mesh] OR “Insecticides”[Mesh] OR “Rodenticides”[Mesh] OR “farming” OR “rural living” OR “well water”) AND “Risk”. Similar search terms were used for EMBASE and Web of Science. References from eligible articles were also examined for additional studies. We conducted our meta-analysis according to the PRISMA checklists and followed the relevant guidelines^36^.

### Study selection

We selected studies on the basis of the following criteria: cohort or case-control study design, the exposure of interest being pesticides and the outcome being the incidence of AD, and articles that reported at least one effect size with 95% CI relating pesticide exposure to AD or enough data to calculate effect size. Serum pesticide exposure data were excluded, as these data could not be meta-analysed because the results were reported in different formats. Studies that utilized mortality data for ascertainment of AD were also excluded, as AD may be frequently underreported on death certificates.

### Data extraction

Two investigators (D.Y. and Y.Z.) extracted the data and evaluated the eligibility of potential studies according to the guidelines for meta-analysis^37^. Extracted information included study characteristics, sample size, length of follow-up period in cohort study, diagnostic criteria of AD, exposure assessment, adjustment variables, effect sizes, and 95% CIs. In individual studies, the adjusted effect sizes were priority selected for the meta-analysis. In order to assess the impact of confounders on the causal relationship between AD and pesticide exposure, a special effort was made to extract crude (unadjusted for confounders by analysis models or in study designs) and adjusted effect sizes separately.

### Quality assessment

The Newcastle-Ottawa Scale^38^ was used independently by two investigators to assess the quality of each publication. It assigns a maximum of 9 points to studies of the highest quality according to 3 parameters: selection (4 points), comparability (2 points), and exposure (case-control studies) or outcome (cohort studies; 3 points). Low, moderate, and high quality studies were assigned scores of 0–3, 4–6, and 7–9, respectively. Any discrepancies were addressed by a joint re-evaluation (by L.L. and H.Y.) of the study.

### Data analysis

We chose ORs as a common measure to assess the relationship between pesticides exposure and AD because when the outcome is rare, ORs, hazard ratios (HRs), and relative risks (RRs) provide similar estimates of risk^39^. Possible heterogeneity among studies was investigated using the Cochran *Q* and *I^2^* statistics^40^. A low *P* value for the Cochran *Q* statistic indicates a significant level of heterogeneity. The *I^2^* metric describes the percentage of total variation among studies that is due to heterogeneity rather than chance^34^,^41^. Low, moderate, and high degrees of *I^2^* values were considered to be 25%, 50%, and 75%^34^, respectively. A fixed-effect model is applied when heterogeneity is negligible; otherwise, a random-effect model is used^34^. The quality-effect model^42^ (based on quality scores) was also employed, and we compared the results of the quality-effect model analysis with those from the random-effect and fixed-effect models.

To explore the potential effects of specific study characteristics on the association between pesticide exposure and AD, subgroup analyses were conducted according to source of controls, study quality, methods of exposure assessment, age, and adjustments. Publication bias was evaluated by Egger’s test and Begg’s test^43^,^44^. Sensitivity analyses were used to evaluate the sensitivity of our results.

Except for quality-effect modelling (conducted with MetaXL version 2.2 software), all statistical analyses were conducted with Stata 12.0 software (StataCorp). All reported probabilities (*P* values) were two-sided, and *P* < 0.05 was considered statistically significant.

## Additional Information

**How to cite this article**: Yan, D. *et al*. Pesticides exposure and risk of Alzheimer’s disease: a systematic review and meta-analysis. *Sci. Rep.* **6**, 32222; doi: 10.1038/srep32222 (2016).

## Supplementary Material

Supplementary Information

Supplementary Figure S1

Supplementary Table S1

## Figures and Tables

**Table 1:** Selected characteristics of studies on pesticide exposure and Alzheimer’s disease.

Study	Study design	Country	Exposure assessment	AD diagnosis criteria	Case	Control	Adjustments	Remarks
Tyas *et al*.^17^	Cohort	Canada	Prospectively, self-report ever/never occupational exposure	The 3MS was used for cognitive impairment and then NINCDS-ADRDA was used for AD diagnosis	36 patients with AD after 5-y follow-up. Mean age 79.8y.	Baseline, 1,039 persons. After excluding ineligible participants, 658 controls left. Mean age 73.7y	Age, education, sex	Crude ES calculated from reported numbers
Baldi *et al*.^18^	Cohort	France	Prospectively, self-report ever/never occupational exposure	The 3MS was used for cognitive impairment and then NINCDS-ADRDA was used for AD diagnosis	96 patients with AD after 5-y follow-up. Mean age 79.2y	Baseline, 1507 persons who were >65 y of age in specific area. Mean age 78.4y	Smoking, education	The ES composed the males and females
Hayden *et al*.^12^	Cohort	USA	Prospectively, self-report ever/never occupational exposure	The 3MS was used for cognitive impairment and then NINCDS-ADRDA was used for AD diagnosis	344 patients with AD after 7.2-y follow-up. Mean age 74.1y	Baseline, 3,048 persons. Mean age 74.5y	Age, sex, education, Mini-Mental State Examination score, APOEε4 status	—
Gun *et al*.^19^	Case-control (medical practice based)	Australia	Retrospectively, proxy reports job history and code into JEM	The NINCDS-ADRDA was used for AD diagnosis	170 patients with AD. Mean age of men was 77.4y. Mean age of women was 77.1y. Response rate, 100%.	170 controls. Mean age of men was 77.1y. Mean age of women was 76.7y, Response rate, 100%.	—	—
CSHA *et al*.^20^	Case-control (population- based)	Canada	Retrospectively, proxy reports risk factor information	The 3MS was used for cognitive impairment and then NINCDS-ADRDA was used for AD diagnosis	258 patients with AD, within 3y of diagnosis. Mean age 84.1y. Response rate, 83.9%.	353 controls. Mean age 79y, Response rate, 89%	Age, sex, education, residence in community or institution	Crude ES calculated from reported numbers
Gauthier *et al*.^21^	Case-control (population- based)	Canada	Retrospectively, proxy reports job history and code into JEM	The 3MS was used for cognitive impairment and then NINCDS-ADRDA was used for AD diagnosis.	68 patients with AD, age >70y.	68 controls, age >70y.	Education, family history, APOEε4 status.	—
French *et al*.^22^	Case-control (hospital control)	USA	Retrospectively, proxy reports risk factor information.	No details were reported regarding AD diagnosis.	78 male patients with AD, age <60y, 12.9%; age 60–79y, 44.8%; age >80y, 42.3%.	76 hospital controls, age <60y, 13.1%; age 60–79y, 42.1%; age >80y, 44.8%.	—	—

Abbreviations: AD, Alzheimer’s disease; CI, confidence interval; CSHA, Canadian Study of Health and Aging; ES, effect size; JEM, job exposure matrix; 3MS, the Modified Mini-Mental State Examination; NINCDS-ADRDA, the National Institute of Neurological and Communicative Disorders and Stroke–Alzheimer’s Disease and Related Disorders Association.

**Table 2:** Alzheimer’s disease and pesticide exposure: Summary ES after stratification of all studies.

Analysis category	No. of studies	Fixed effects model		Heterogeneity statistics		Publication bias (*P* Value)	
		OR	95% CI	*I^2^* Index (%)	*P* Value	Egger	Begg
Pesticide exposure	7	1.34	1.08, 1.67	0	0.88	0.66	0.76
Crude ES studies	7	1.14	0.94, 1.38	0	0.50	0.95	1.00
Adjusted ES studies	5	1.37	1.09, 1.71	0	0.92	0.55	0.81

Abbreviations: CI, confidence interval; ES, effect size; N, number; OR, odds ratio.
