# Supplementary material for: Pesticide exposure and risk of Alzheimer’s disease: a systematic review and meta-analysis
Source: Sci Rep. 2016 Sep 1;6:32222. doi: 10.1038/srep32222 (PMC5007474; doi:10.1038/srep32222)
Supplement: Supplementary Figure S1 [file srep32222-s2.pdf]

**Supplementary Figure S1**

# Pesticides exposure and risk of Alzheimer's disease: a systematic review and meta-analysis

**Authors**

Dandan Yan, Yunjian Zhang, Liegang Liu, Hong Yan *

Dandan Yan, department of Health Toxicology, MOE Key Lab of Environment and Health, School of Public Health, Tongji Medical College, Huazhong University of Science and Technology, 13 Hangkong-Road, Wuhan, 430030, PR China.

E-mail:460316065@qq.com

Yunjian Zhang, department of Huazhong Univ Sci & Technol, Tongji Med Coll, Union Hosp, Dept Neurol, Wuhan 430030, Peoples R China

E-mail: Zhangyunjian66@126.com

Liegang Liu, department of Nutrition and Food Hygiene, Hubei Key Laboratory of Food Nutrition and Safety, Tongji Medical College, Huazhong University of Science and Technology, 13 Hangkong-Road, Wuhan, 430030, PR China.

E-mail: liuliegang@mails.tjmu.edu.cn

***Corresponding author:**

Hong Yan: Department of Health Toxicology, MOE Key Lab of Environment and Health, School of Public Health, Tongji Medical College, Huazhong University of Science and Technology, 13 Hangkong-Road, Wuhan, 430030, PR China.

**E-mail:** yanhong@mails.tjmu.edu.cn (H. Yan)

Phone: +86-27-83692720; Fax: +86-27-83692333.

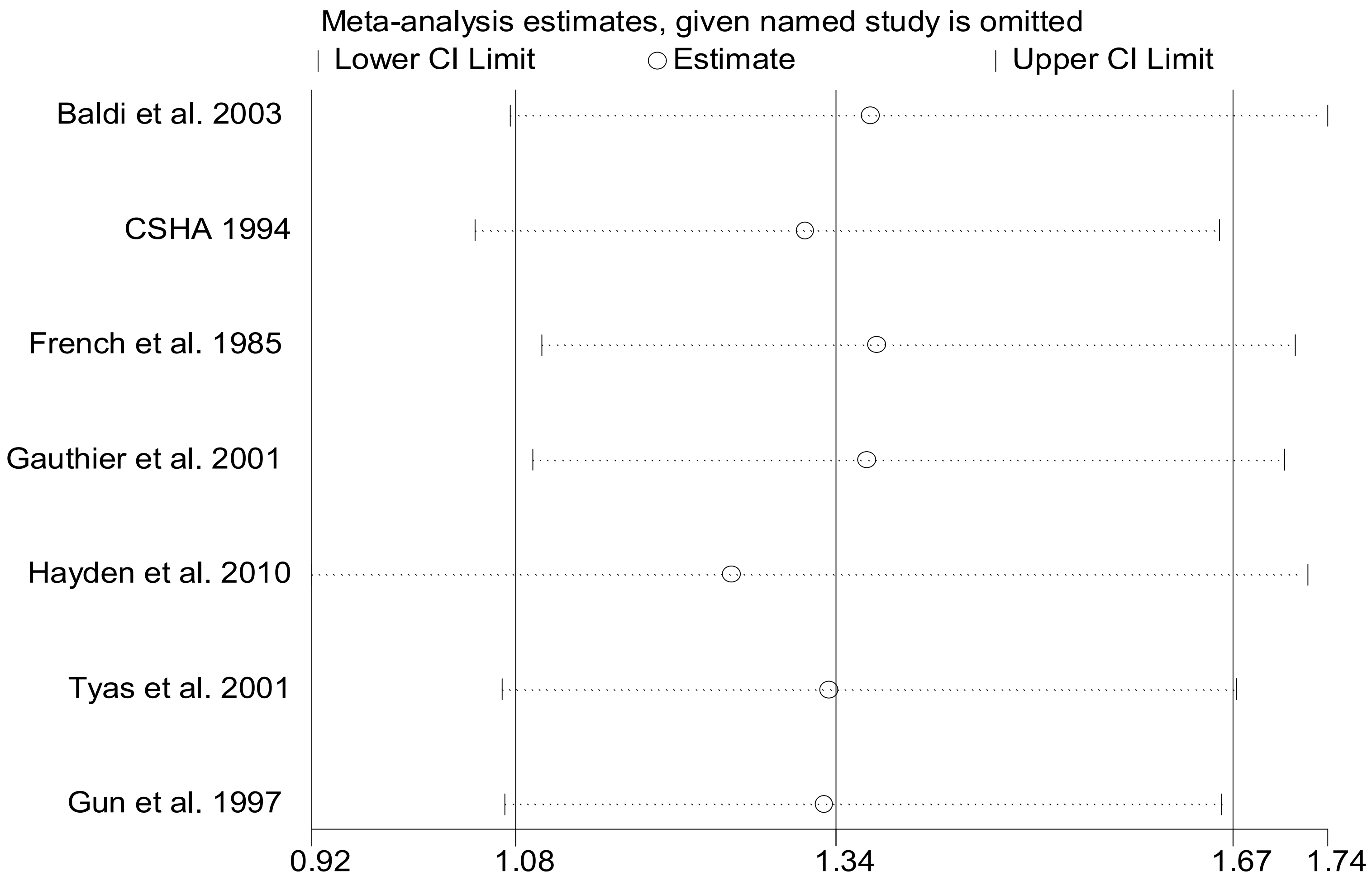

Meta-analysis estimates, given named study is omitted
Lower CI Limit
Estimate
Upper CI Limit
Baldi et al. 2003
CSHA 1994
French et al. 1985
Gauthier et al. 2001
Hayden et al. 2010
Tyas et al. 2001
Gun et al. 1997
0.92
1.08
1.34
1.67
1.74

**Supplementary Figure S1: Results of sensitivity analysis using leave-one-out method.**

Figure legend: The circles and the horizontal lines represent the ORs and 95% CIs after omitting studies in turn. The vertical dashed lines show the OR of 1.08 and 1.67.
