# Supplementary material for: Pesticide exposure and risk of Alzheimer’s disease: a systematic review and meta-analysis
Source: Sci Rep. 2016 Sep 1;6:32222. doi: 10.1038/srep32222 (PMC5007474; doi:10.1038/srep32222)
Supplement: Supplementary Table S1 [file srep32222-s3.pdf]

Supplementary Table S1

Pesticides exposure and risk of Alzheimer's disease: a systematic review and meta-analysis

Authors

Dandan Yan, Yunjian Zhang, Liegang Liu, Hong Yan *

Dandan Yan, department of Health Toxicology, MOE Key Lab of Environment and Health, School of Public Health, Tongji Medical College, Huazhong University of Science and Technology, 13 Hangkong-Road, Wuhan, 430030, PR China.

E-mail:460316065@qq.com

Yunjian Zhang, department of Huazhong Univ Sci & Technol, Tongji Med Coll, Union Hosp, Dept Neurol, Wuhan 430030, Peoples R China

E-mail: Zhangyunjian66@126.com

Liegang Liu, department of Nutrition and Food Hygiene, Hubei Key Laboratory of Food Nutrition and Safety, Tongji Medical College, Huazhong University of Science and Technology, 13 Hangkong-Road, Wuhan, 430030, PR China.

E-mail: liuliegang@mails.tjmu.edu.cn

***Corresponding author:**

Hong Yan: Department of Health Toxicology, MOE Key Lab of Environment and Health, School of Public Health, Tongji Medical College, Huazhong University of Science and Technology, 13 Hangkong-Road, Wuhan, 430030, PR China.

E-mail: yanhong@mails.tjmu.edu.cn (H. Yan)

Phone: +86-27-83692720; Fax: +86-27-83692333.

Table S1. Quality assessment of the included studies on pesticide exposure and risk of Alzheimer's disease.

Table S1A Quality assessment of the cohort studies on pesticide exposure and Alzheimer's disease.

	Selection				Comparability		Exposure			
	1	2	3	4	5A	5B	6	7	8	
Study	Indicates exposed cohort truly representative	Non-exposure cohort drawn from the same community	Ascertainment of exposure by structured interview	Outcome of interest not present at start	Cohorts comparable on basis of age, sex, and education level	Cohorts comparable on other factor(s)	Quality of outcome assessment	Enough follow-up period for outcome to occur	Complete accounting for cohorts	Overall quality score
Baldi et al. 2003	1	1	0	1	1	0	0	1	1	6
Tyas et al. 2001	1	1	1	1	1	0	1	1	1	8
Hayden et al. 2010	1	1	1	1	1	1	0	1	1	8

The study quality was assessed according to the Newcastle Ottawa Quality assessment scale for cohort studies. This scale awards a maximum of 9 points to each study: 4 for selection, 2 for comparability, and 3 for exposure (for cohort study). 1 = "Yes", 0 = "No", "Unable to determine" or "Not available". For cohort studies, 1, indicates exposed cohort truly representative; 2, non-exposed cohort drawn from the same community; 3, ascertainment of exposure by JEM; 4, outcome of interest not present at start; 5A, cohorts comparable on basis of age, sex, and education level; 5B, cohorts comparable on other factor(s); 6, quality of outcome assessment; 7, enough follow-up period for outcome to occur; and 8, complete accounting for cohorts.

Abbreviations: AD, Alzheimer's disease

Table S1B Quality assessment of the case-control studies on pesticide exposure and Alzheimer's disease.

	Selection				Comparability		Exposure			
	1	2	3	4	5A	5B	6	7	8	
Study	Indicates case independently validated	Case are representative of population	Community controls	Controls have no AD	Study controls for age or sex or education level	Study controls for additional factor(s)	Ascertainment of exposure by blinded interview	Same method of ascertainment used for case and controls	Non-response rate the same for cases and controls	Overall quality score
Gun et al. 1997	1	1	0	1	0	0	0	1	1	5
CSHA et al. 1994	1	1	1	1	1	0	0	1	1	7
Gauthier et al. 2001	1	1	1	1	1	1	0	1	1	8
French et al. 1985	1	1	0	1	0	0	0	1	1	5

The study quality was assessed according to the Newcastle Ottawa Quality assessment scale for case-control studies. This scale awards a maximum of 9 points to each study: 4 for selection, 2 for comparability, and 3 for assessment of exposure (for case-control study). 1 = "Yes", 0 = "No", "Unable to determine" or "Not available". For case-control studies, 1, indicates cases independently validated; 2, cases are representative of population; 3, community or population based controls; 4, controls have no Alzheimer's disease; 5A, study controls for age or sex or education level; 5B, study controls for additional factor(s); 6, ascertainment of exposure by blinded interview; 7, same method of ascertainment used for cases and controls; and 8, non-response rate the same for cases and controls.

Abbreviations: AD, Alzheimer's disease; CSHA, Canadian Study of Health and Aging.
